# Highly Efficient and Low-Temperature Preparation of Plate-Like ZrB_2_-SiC Powders by a Molten-Salt and Microwave-Modified Boro/Carbothermal Reduction Method

**DOI:** 10.3390/ma11101811

**Published:** 2018-09-24

**Authors:** Yuan Zeng, Feng Liang, Jianghao Liu, Jun Zhang, Haijun Zhang, Shaowei Zhang

**Affiliations:** 1 The State Key Laboratory of Refractories and Metallurgy, Wuhan University of Science and Technology, Wuhan 430081, China; 15697180127@163.com (Y.Z.); liangfengref@wust.edu.cn (F.L.); kd_zhangjun@163.com (J.Z.); 2 College of Engineering, Mathematics and Physical Sciences, University of Exeter, Exeter EX4 4QF, UK; S.Zhang@exeter.ac.uk

**Keywords:** ZrB_2_-SiC powders, plate-like morphology, microwave heating, molten-salt synthesis, boro/carbothermal reduction

## Abstract

To address the various shortcomings of a high material cost, energy-intensive temperature conditions and ultra-low efficiency of the conventional boro/carbothermal reduction method for the industrial preparation of ZrB_2_-SiC powders, a novel molten-salt and microwave-modified boro/carbothermal reduction method (MSM-BCTR) was developed to synthesize ZrB_2_-SiC powders. As a result, phase pure ZrB_2_-SiC powders can be obtained by firing low-cost zircon (ZrSiO_4_), amorphous carbon (C), and boron carbide (B_4_C) at a reduced temperature of 1200 °C for only 20 min. Such processing conditions are remarkably milder than not only that required for conventional boro/carbothermal reduction method to prepare phase pure ZrB_2_ or ZrB_2_-SiC powders (firing temperature of above 1500 °C and dwelling time of at least several hours), but also that even with costly active metals (e.g., Mg and Al). More importantly, the as-obtained ZrB_2_ particles had a single crystalline nature and well-defined plate-like morphology, which is believed to be favorable for enhancing the mechanical properties, especially toughness of their bulk counterpart. The achievement of a highly-efficient preparation of such high-quality ZrB_2_-SiC powders at a reduced temperature should be mainly attributed to the specific molten-salt and microwave-modified boro/carbothermal reduction method.

## 1. Introduction

Due to its high melting point and hardness, excellent erosion resistance, good thermal/electrical conductivities, and outstanding oxidation resistance, ZrB_2_-SiC attracted considerable attention in versatile ultra-high-temperature structural applications, including rocket propulsion, cutting-edge space vehicles, and hypersonic flight [1,2,3,4,5].

In the mass preparation of high-performance ZrB_2_-SiC ceramics, an energy saving, large-scale, and efficient preparation of high-quality ZrB_2_-SiC powders is in high demand. Nowadays, the industrial production of ZrB_2_-SiC powders mainly employs the boro/carbothermal reduction (BCTR) approach, with ZrO_2_ and SiO_2_ as Zr and Si as precursors [6,7,8,9]. However, this method suffers from several significant disadvantages, such as the requirement of a high processing temperature (1500–1700 °C) and long soaking time (at least several hours) as well as a high agglomeration degree, low purity, and poor sinterability of powder product. Generally speaking, these shortcomings of the conventional BCTR method are due to the following two reasons: (1) the poor reactivity of commercial ZrO_2_ and SiO_2_ raw materials, resulting in the inefficiency of synthetic process; and (2) the intrinsic requirements for strict temperature conditions and a long soaking time, and phase pure ZrB_2_-SiC powders with a well-defined morphology and textured structure are difficult to prepare by the conventional BCTR method. Therefore, a modification of the conventional method by an advanced synthetic technique, and using high-activity and low-price raw materials to prepare ZrB_2_-SiC powders at milder temperature conditions, is in high demand.

Zircon (ZrSiO_4_), a low-cost and high-abundance material, intended to decompose into highly active ZrO_2_ and SiO_2_ in situ, has been proved to be favorable for enhancing the synthetic reactions of ZrB_2_-SiC powders [10,11]. Moreover, the theoretical phase composition of the final product, resulting from ZrSiO_4_, was ZrB_2_-26 wt % SiC, which was among the as-reported optimal composition range for the preparation of the bulk counterpart, with promising mechanical properties and oxidation resistance [12,13]. As a result, zircon had been widely adopted in the preparation of ZrB_2_-SiC powders. For example, Krishnarao [14] prepared ZrB_2_-SiC powders by firing a mixture of zircon, C and B_4_C, although a high processing temperature of 1600 °C was required. In our previous work [15], zircon, C, and B_2_O_3_ were used to prepare uniformly distributed ZrB_2_-SiC powders at the relatively lower temperature of 1300 °C for 3 h via a novel microwave-modified BCTR approach. However, it should be emphasized that it is still a challenging task to control the morphology of the ZrB_2_-SiC powders resulting from the conventional reduction process.

In this respect, several advanced techniques employing microwave and molten salt were utilized to modify the conventional reduction method for synthesizing ZrB_2_-SiC powder [5,15,16]. On the one hand, molten salt is capable of providing the expected synthetic reactions with a liquid medium, thus facilitating homogeneous mixing, accelerating the diffusion of reactants, and enhancing the synthesis and crystal growth of products to form a well-defined anisotropic morphology [17,18]. On the other hand, microwave heating is capable of uniformly heating the raw materials, thus greatly accelerating the overall synthetic reaction [19,20]. More importantly, it is reported that a microwave is capable of inducing the rapid nucleation of the product, crystallite, so as to accelerate the crystal growth and enhance the crystallization degree, thereby facilitating the control of the crystal size and morphology of the final product [21,22,23,24]. For example, Cho [25] reported that ZnO nano-rods and nano-plates with a high crystallization degree could be obtained using a microwave heating technique, which can be attributed to the control of crystal growth and dissolution rates in specific directions. Liu [26] claimed that the crystallization behavior of NaHSO_4_ was closely related to microwave heating, which facilitated the crystal growth and finally controlled the crystal size of the product. To sum up, microwave heating and a molten-salt medium were favorable for accelerating the preparation of ZrB_2_-SiC powder, with a textured morphology at a reduced temperature.

In this work, by using low-cost zircon (ZrSiO_4_), ZrB_2_ (plate-like)-SiC powders were prepared in a highly-efficient manner by a molten-salt and microwave-co-modified boro/carbothermal reduction method (MSM-BCTR) at a reduced temperature. The effects of various processing parameters—including firing temperature, microwave heating/molten salt medium, salt/reactant weight ratio, and B_4_C amounts on the synthesis of textured ZrB_2_-SiC powders—were systematically investigated. More interestingly, the as-synthesized ZrB_2_ particles had a well-defined plate-like morphology and single crystalline nature, which is believed to be favorable for enhancing the mechanical properties, especially toughness, of their bulk counterpart.

## 2. Experimental Procedures

### 2.1. Raw Materials

Zircon (ZrSiO_4_, purities > 95.0%, Bodi Chem. Co. Ltd., Tianjin, China), boron carbide (B_4_C, purity > 95.0%, Mudanjiang Jingangzuan Boron Carbide Co. Ltd., Mudanjiang, China), and amorphous carbon (C, Co. Ltd., Shanghai, China, *d_50_* = 37.0 μm) were used as raw materials. Eutectic salts of NaCl and KCl (purities > 99.9%, Bodi Chem. Co. Ltd., Tianjin, China) were used as a reaction medium. These chemicals were used directly, without further purification.

In present paper, the target reactions of synthesizing ZrB_2_-SiC powders were presented as follows.

ZrSiO_4(s)_ = ZrO_2(s)_ + SiO_2(s)_ (1)

2ZrO_2(s)_ + B_4_C_(s)_ + 3C_(s)_ = 2ZrB_2(s)_ + 4CO_(g)_ (2)

SiO_2(s)_ + 3C_(s)_ = SiC_(s)_ + 2CO_(g)_ (3)

2ZrO_2(s)_ + 2SiO_2(s)_ + B_4_C_(s)_ + 9C_(s)_ = 2ZrB_2(s)_ + 8CO_(g)_ + 2SiC_(s)_ (4)

In order to minimize residual carbon, which may seriously degrade the sinterability of the ZrB_2_-SiC powder product, an addition amount of carbon was fixed at a stoichiometric ratio, according to the expected Reaction (4). Moreover, various excess amounts of B_4_C (40–60 mol % excess) were used to compensate for the volatilization loss of the boron source at a high temperature. Salts were mixed with reactants in various weight ratios of 2.0, 1.0, and 0.5, while the weight ratio of NaCl and KCl was fixed at 1.0:1.0. The processing conditions and batch compositions of samples are listed in Table 1.

### 2.2. Methodologies

**Figure 1:**
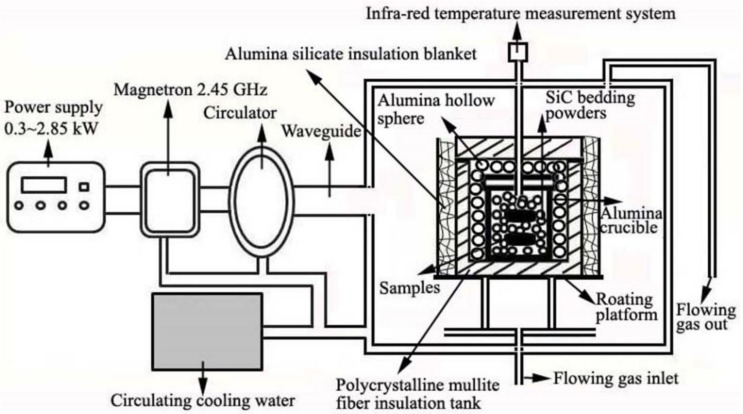
Schematic diagram of the microwave heating furnace.

In a typical MSM-BCTR process, reactant and salt mediums (as presented in Table 1) were firstly mixed in a corundum crucible by hand and then contained by a columnar saggar. The free space between the corundum and saggar was filled with green SiC particles, owing to their superior microwave absorbability and excellent thermal conductivity. Afterwards, the saggar was positioned in the center of a microwave furnace (HAMiLab-V3000, 3 kW, 2.45 GHz, Changsha Longtech Co. Ltd., China), whose temperature was monitored by an infrared thermometer (Yongtai, Xian, Chian), vertically pointing to the green SiC particles. The schematic diagram of the microwave heating system was shown in Figure 1. Then, samples were heated to 1100–1250 °C, at a constant heating rate of 10 °C/min and held for 0–20 min in flowing argon before naturally cooling to room temperature. Finally, the as-obtained powders were repeatedly washed with hot water (80 °C) and to remove residual salt, before drying overnight at 80 °C in a vacuum oven.

Crystalline phases of the as-obtained powders were identified by X-ray diffraction (Xpertpro, PHILIPS, Hillsboro, The Netherlands), with the spectra ranging from 10° to 90° (2θ), a scanning rate of 2°/min and Cuk*α* radiation (*λ* = 0.1542 nm). ICDD cards no. 75-0254, 73-1708, 74-1200, and 83-1374 were used to identify ZrB_2_, SiC, ZrO_2_, and ZrSiO_4_. A field-emission scanning electron microscope (FE-SEM, Nova400NanoSEM, PHILIPS, Amsterdam The Netherlands, 15 kV), equipped with an energy dispersive spectrometer (EDS, IET 200, Oxford, UK) and transmission electron microscope (TEM, JEM-2100UHRSTEM, JEOL, Akishima, Tokyo, Japan, 200 kV), was used to characterize the microstructures and morphologies of the as-obtained ZrB_2_-SiC powders.

## 3. Results and Discussion

### 3.1. Effect of Firing Temperature on the Synthesis of ZrB_2_-SiC Powders

**Figure 2:**
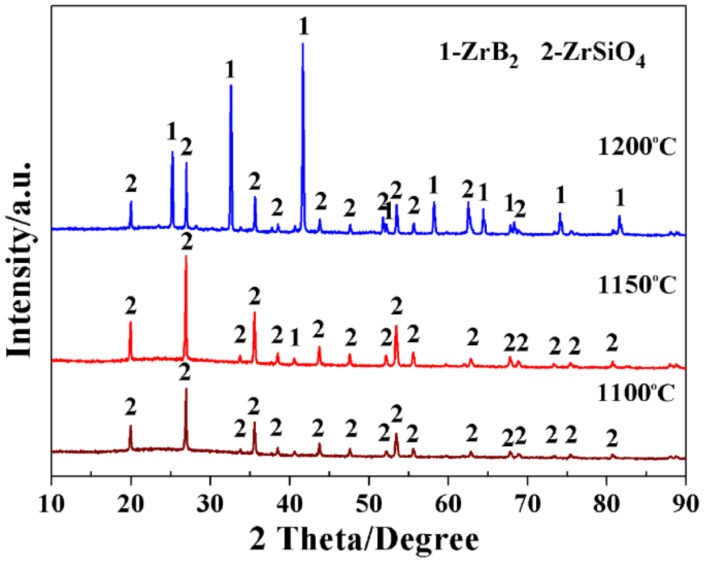
XRD patterns of the samples prepared by the MSM-BCTR method at 1100–1200 °C, without soaking period.

XRD patterns of the samples prepared at different temperatures, with the identical batch composition of reactants (ZrSiO_4_/C = 1.0/4.5, 60 mol % excess B_4_C, and weight ratio between salt medium/reactants = 2.0), were presented in Figure 2. For the sample (MB-1) prepared at 1100 °C, all the visible diffraction peaks belonged to ZrSiO_4_, implying the decomposition of zircon (Reaction (1)) had not yet occurred. Upon increasing the temperature to 1150 °C (MB-2), some peaks indexing to the ZrB_2_ phase appeared, indicating that the onset temperature of synthesizing ZrB_2_ was close to 1150 °C, which was dramatically lower than that of the conventional method [27,28]. Besides, neither SiO_2_ nor ZrO_2_ was detected in this sample, suggesting that Reaction (2) and Reaction (3) were so efficient that intermediate products (ZrO_2_ and SiO_2_) were not detected. On increasing the temperature to 1200 °C (MB-3), the intensities of ZrB_2_ peaks evidently increased, while those of the ZrSiO_4_ peaks accordingly decreased, indicating that the increase in firing temperature had a positive effect on facilitating the synthesis of target phases.

**Figure 3:**
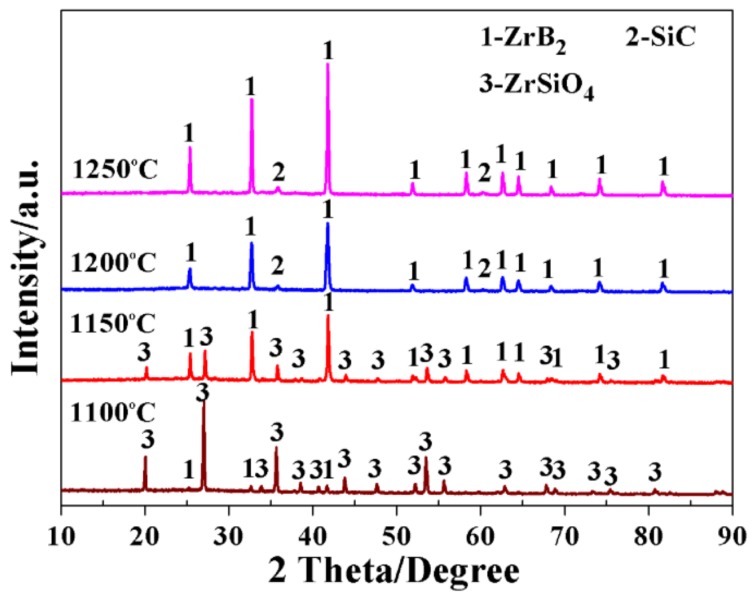
XRD patterns of the samples resulting from the MSM-BCTR method at 1100–1200 °C, with a soaking time of 20 min.

Subsequently, with the ambition of improving the purity of the powder products, a series of experiments were carried out over the as-optimized temperature range of 1100–1250 °C, with a slightly extended soaking time of 20 min. As shown in Figure 3, the sample obtained at 1100 °C (MB-4) contained large amounts of unreacted ZrSiO_4_ and a minor amount of ZrB_2_, further confirming that the expected reaction that the synthesizing ZrB_2_ was conducted with a low efficiency at this temperature. Upon increasing the temperature to 1150 °C (MB-5), the intensities of ZrB_2_ peaks greatly increased, while those of residual ZrSiO_4_ evidently decreased, suggesting that the expected reactions were effectively accelerated by the rising temperature. Moreover, as the temperature increased to 1200 °C (MB-6), the diffraction peaks indexing to the cubic SiC phase appeared, implying that the onset formation temperature of SiC was close to 1200 °C, which was a bit higher than that of ZrB_2_ under the present condition. Interestingly, only ZrB_2_ and SiC phases were detected in this sample, indicating that synthetic reaction of ZrB_2_-SiC powders had been completed. On further increasing the temperature to 1250 °C (MB-7), no further change in the diffraction peaks of the target phases could be observed, suggesting that further increasing the processing temperature to above 1200 °C was unnecessary in the present work.

It should be emphasized that the preparation conditions (1200 °C/20 min) for phase pure ZrB_2_-SiC powders was almost the lowest according to Table 2, among that reported for synthesizing ZrB_2_ or ZrB_2_-SiC by the methodologies based on thermal-reduction process [5,14,15,27,28,29,30,31,32,33,34,35,36,37,38], not only remarkably milder than that (several hours or more) required for conventional BCTR to prepare phase pure ZrB_2_ or ZrB_2_-SiC powders, but also that even with costly active metals (e.g., Mg and Al) [30,39] or boron [29,31,35] as the additional reducing agent. Such great enhancement to the synthetic result of ZrB_2_-SiC powders should be attributed to the combined effects of microwave heating and molten-salt medium.

**Figure 4:**
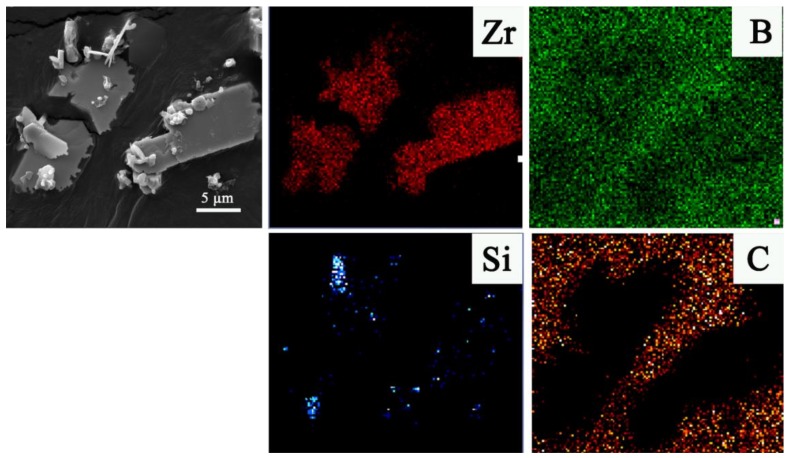
A lower magnification SEM image with EDS mapping of as-prepared ZrB_2_-SiC powders fired at 1250 °C for 20 min with 60 mol % excess B_4_C and a salt-medium/reactant weight ratio of 2.0.

The FE-SEM image presented in Figure 4 showed that there existed well-defined micron-sheets in the final product fired at 1250 °C. As confirmed by EDS mapping, the plate-like particles should be ZrB_2_, which were several micrometers in width and hundreds of nanometers in thickness, and it is believed to be favorable for enhancing the mechanical properties, especially toughness of their bulk counterpart. While those having low crystallinity and amorphous morphology belonged to SiC, it further confirmed that the two phases of ZrB_2_ and SiC co-existed and were homogeneously distributed with each other in the powder product

**Figure 5:**
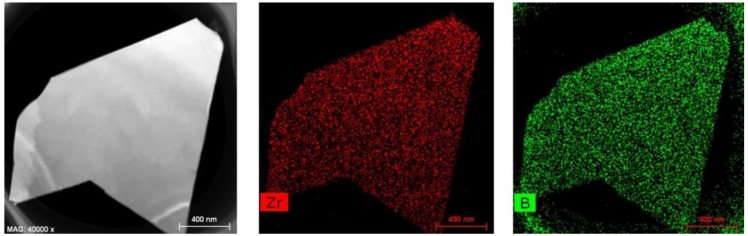
TEM and EDS mapping images of the as-prepared ZrB_2_-SiC composite powders fired at 1250 °C for 20 min.

**Figure 6:**
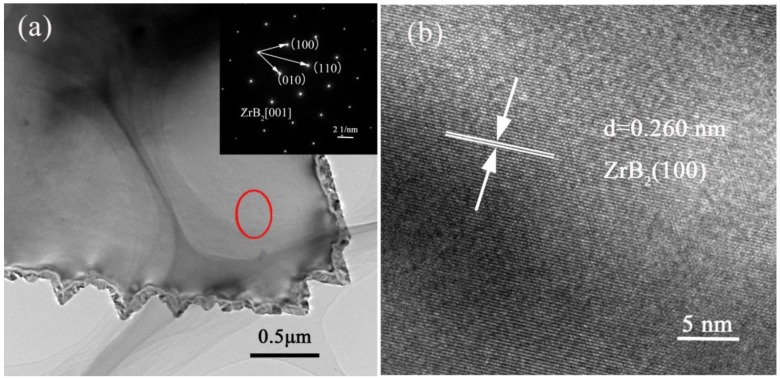
(**a**) Low-resolution TEM image and SAED (inserted in Figure 4**a**), and (**b**) high-resolution TEM image of the as-prepared ZrB_2_-SiC powders fired at 1250 °C for 20 min.

**Figure 7:**
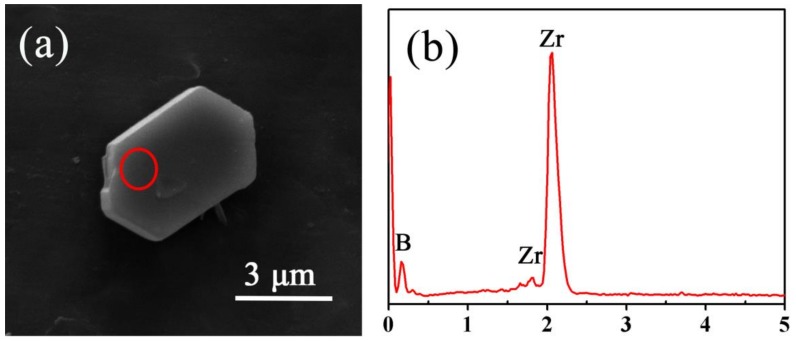
(**a**) SEM image with EDS with EDS (**b**) of as-prepared ZrB_2_-SiC powders fired at 1250 °C for 20 min with 60 mol % excess B_4_C and a salt-medium/reactant weight ratio of 2.0.

TEM and EDS mapping images of as-obtained ZrB_2_-SiC powders (shown in Figure 5) further confirmed the formation of plate-like ZrB_2_. The TEM and SAED results (inserted in Figure 6a) verified that the plate-like particle was single-crystal ZrB_2_, which was fabricated by epitaxial growth along its [001] direction. As shown in the HRTEM image (Figure 6b), specifically in the area marked by the red circle in Figure 6a, the plate-like particles had well-aligned lattice fringes, with a constant interplanar spacing of 0.260 nm, which matched well with that of the (100) interplanar distance of ZrB_2_ crystal. Thus, it can be concluded that ZrB_2_ with a single-crystalline nature and anisotropic plate-like morphology was prepared by the present molten-salt and microwave-co-assisted boro/carbothermal reduction method. The TEM image of a SiC particle was not presented in this paper because it had a low crystallinity and no typical morphology. Moreover, as confirmed by Figure 7, the as-formed ZrB_2_ plate generally had a near-hexagonal-shaped morphology and was distributed uniformly in the final product.

### 3.2. Effect of a Microwave Heating/Molten-Salt Medium on the Synthesis of ZrB_2_-SiC Powders

**Figure 8:**
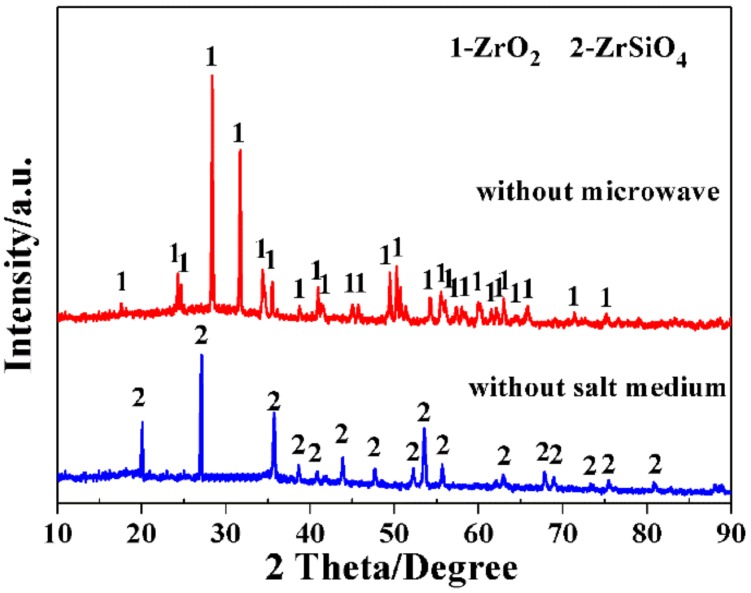
XRD patterns of the samples prepared at 1200 °C/20 min without either microwave heating or a molten-salt medium condition.

The achievement of a low-temperature rapid synthesis of ZrB_2_-SiC with a plate-like morphology and single-crystalline nature was closely related to the specific MSM-BCTR conditions, characterized by microwave heating and a molten-salt medium. To further clarify their effects on the synthesis of ZrB_2_-SiC powders, the subsequent experiments were carried out under the as-optimized MSM-BCTR conditions, without either a microwave or molten-salt medium for comparison. As presented in Figure 8, for the sample (MB-10) prepared by a microwave-assisted BCTR method without molten salt, only raw ZrSiO_4_ existed, implying no occurrence of Reaction (1), which was consistent with the as-reported results that the decomposition of ZrSiO_4_ required a temperature as high as 1600 °C [40,41]. On the other hand, in the contrasting case, without microwave heating (MB-11), only ZrO_2_ peaks existed, indicating that ZrSiO_4_ had been completely decomposed, and the absence of SiO2 should be attributed to its poor crystallinity. This result verified that the molten-salt medium greatly enhanced the decomposition of ZrSiO_4_ and thereby accelerated the overall synthetic reactions of ZrB_2_-SiC powders.

### 3.3. Effect of B_4_C Addition Amount on the Synthesis of ZrB_2_-SiC Powders

**Figure 9:**
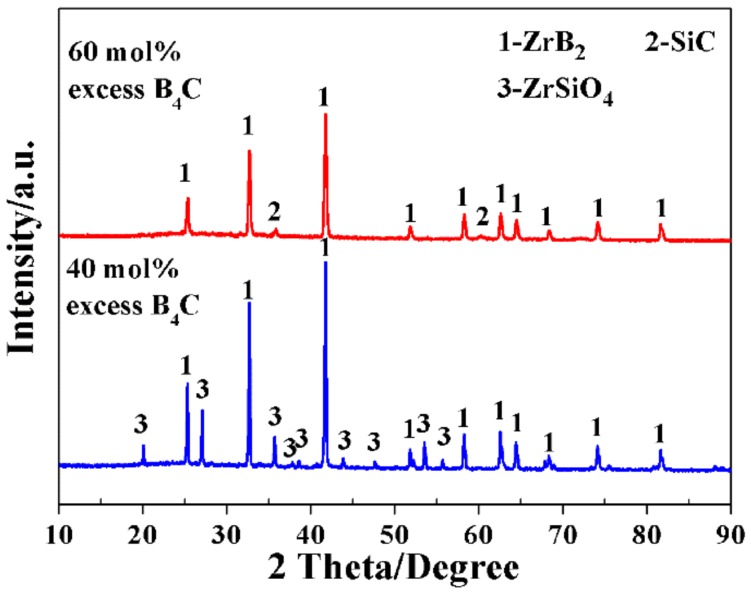
XRD patterns of the samples prepared at 1200 °C/20 min with 40 mol % and 60 mol % excess B_4_C in raw materials.

B_4_C played a dual role of boron source and reducing agent in the synthesis of ZrB_2_-SiC powders. To clarify the effect of additional amounts of B_4_C on the synthesis of ZrB_2_-SiC powder, the following experiments were conducted. As shown in Figure 9, phase pure ZrB_2_-SiC powders were obtained for the sample with 60 mol % excess B_4_C. On decreasing the excess additional amounts of B_4_C to 40 mol %, not only unreacted ZrSiO_4_ appeared, but also the expected SiC disappeared, indicating that certain excess amounts of B_4_C are necessary to compensate for the volatilization loss of intermediate B_2_O_3_ in order to synthesize phase pure plate-like ZrB_2_-SiC powders.

### 3.4. Effect of Salt/Reactant Weight Ratio on the Synthesis of ZrB_2_-SiC Powders

**Figure 10:**
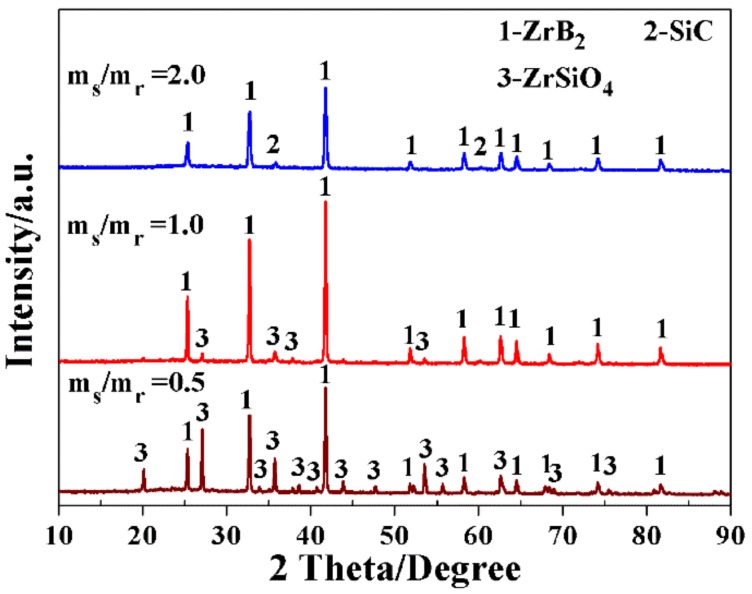
XRD patterns of the samples resulting from the MSM-BCTR method at 1200 °C/20 min, with various salt/reactant weight ratios of 0.5, 1.0, and 2.0, respectively.

As discussed above, the molten-salt medium played an essential role in accelerating the expected synthetic reactions. To elucidate the influence of the amounts of the molten-salt medium on the synthesis of ZrB_2_-SiC powders, the following samples were prepared with various salt/reactant weight ratios of 0.5, 1.0, and 2.0. As shown in Figure 10, the intensities of ZrB_2_ peaks decreased, and the characteristic peaks, indexing to SiC, disappeared, while those of ZrSiO_4_ peaks accordingly increased as the salt/reactant weight ratio decreased. On further decreasing the ratio to 0.5, a larger amount of unreacted ZrSiO_4_ existed in the final product. It can be confirmed that the synthetic reactions were greatly enhanced by appropriate amounts of the molten-salt medium, while an insufficient amount of the molten-salt medium would hinder the rapid conduct of the target reactions.

**Figure 11:**
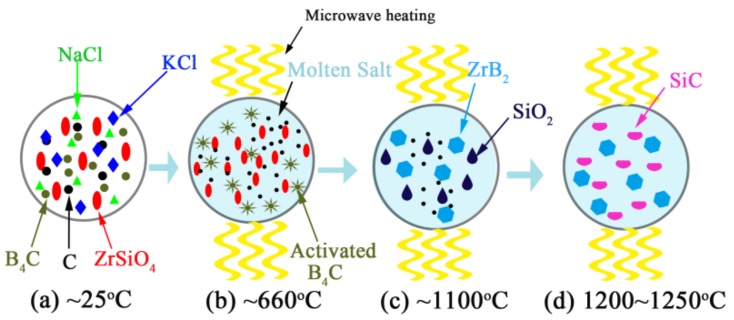
A schematic diagram of the MSM-BCTR process of ZrB_2_-SiC powders.

Based on the results presented and discussed above, a possible mechanism of the MSM-BCTR process was proposed and described schematically as follows (Figure 11): (1) ZrSiO_4_, C, and B_4_C were partly dissolved and homogeneously mixed in the molten-salt medium at the atomic level [42]. Moreover, B_4_C was preferentially activated and heated due to its excellent microwave absorption under the microwave condition (Figure 11b). (2) Consequently, ZrSiO_4_ was rapidly decomposed into SiO_2_ and ZrO_2_, in which ZrO_2_ immediately reacted with B_4_C and C, forming ZrB_2_, when fired at 1100 °C, according to Reactions (1) and (2) under the existence of a molten-salt medium (Figure 11c). (3) Upon increasing the temperature to 1200 °C, SiC was formed via Reaction (3), and once the ZrB_2_ and SiC crystals were oversaturated in molten salt, they started to precipitate from the medium (Figure 11d). The precipitation of ZrB_2_ and SiC crystals from the oversaturated salt led to the further dissolution of the starting materials and the occurrence of a synthetic reaction. These processes were repeated again and again until the target reactions were completely accomplished and phase pure ZrB_2_ and SiC were obtained (Figure 11d).

To sum up, the optimal processing conditions for synthesizing ZrB_2_-SiC powder via the present MSM-BCTR method was 1200 °C/20 min, with 60 mol % excess B_4_C and a salt/reactant weight ratio of 2.0. The temperature conditions were significant milder than those required by the conventional BCTR approach, and this achievement should mainly be ascribed to the combination effect of the synthesis of microwave heating and a molten-salt medium.

## 4. Conclusions

Phase-pure ZrB_2_-SiC powders with a single-crystalline nature and plate-like morphology were prepared through a MSM-BCTR method, using economical zircon, B_4_C, and amorphous C as starting materials, and NaCl-KCl as a reaction medium. ZrSiO_4_ was completely converted into ZrB_2_ and SiC at 1200 °C/20 min using 60 mol % excess B_4_C, with a weight ratio of molten salt medium/reactant of 2.0. Moreover, as-prepared ZrB_2_ particles demonstrated a plate-like single-crystal structure, several micrometers in width and hundreds of nanometers in thickness, and it grew along the [100] direction. The efficient synthesis of phase pure ZrB_2_-SiC powders at such a milder condition than that of the conventional BCTR method was attributed to the synergistic effect of molten-salt and microwave heating.

## Figures and Tables

**Table 1:** Batch compositions and processing conditions for the preparation of ZrB_2_-SiC powders

Sample Number	Molar Ratio			Heating Mode	Temperature (°C)	Soaking Time (min)	Salt Medium	Weight Ratio of Salt/Reactant
	ZrSiO_4_	B_4_C	C					
MB-1	1.00	0.80	4.50	MWH	1100	0	NaCl/KCl	2.0
MB-2	1.00	0.80	4.50	MWH	1150	0	NaCl/KCl	2.0
MB-3	1.00	0.80	4.50	MWH	1200	0	NaCl/KCl	2.0
MB-4	1.00	0.80	4.50	MWH	1100	20	NaCl/KCl	2.0
MB-5	1.00	0.80	4.50	MWH	1150	20	NaCl/KCl	2.0
MB-6	1.00	0.80	4.50	MWH	1200	20	NaCl/KCl	2.0
MB-7	1.00	0.80	4.50	MWH	1250	20	NaCl/KCl	2.0
MB-8	1.00	0.80	4.50	MWH	1200	20	NaCl/KCl	0.5
MB-9	1.00	0.80	4.50	MWH	1200	20	NaCl/KCl	1.0
MB-10	1.00	0.80	4.50	CH	1200	20	NaCl/KCl	2.0
MB-11	1.00	0.80	4.50	MWH	1200	20	—	2.0
MB-12	1.00	0.70	4.50	MWH	1200	20	NaCl/KCl	2.0

MWH and CH denote microwave heating and conventional heating process, respectively.

**Table 2:** Ingredients, modified techniques, processing conditions, and product morphology of previous literatures on the preparation of ZrB_2_ or ZrB_2_-SiC powders by thermal-reduction-based methodologies

Ref. No.	Product	Raw Materials	Modified Technique	Temperature (°C)	Soaking Time (min)	Atmosphere	Morphology of ZrB_2_
[27]	ZrB_2_	ZrO_2_, BN, C	—	1550	90		
[31]	ZrB_2_	ZrO_2_, B	—	1600	90	Vaccum	
[32]	ZrB_2_	ZrO_2_, B_4_C	—	1250	60	Ar	Bar-like
[33]	ZrB_2_	ZrO_2_, B_4_C, C	—	1500	60	Vaccum	Rod-like
[34]	ZrB_2_	ZrO_2_, B_4_C, C	—	1300	60	Vaccum	Rod
[28]	ZrB_2_	ZrO_2_, B_4_C, B_2_O_3_, C	—	1250	180	Ar	Rod-like
[35]	ZrB_2_	Zr(NO_3_)_3_, B, C	—	1550	120		Plate-like
[29]	ZrB_2_	ZrO_2_, H_3_BO_3_, B	—	1000	120		
[36]	ZrB_2_	ZrO_2_, B_4_C, C	—	1650	60	Vaccum	Columnar
[37]	ZrB_2_-SiC	ZrO_2_, H_3_BO_3_, C, SiC	—	1600	90	Ar	Columnar
[14]	ZrB_2_-SiC	ZrSiO_4_, B_4_C, C	—	1600	90	Ar	
[30]	ZrB_2_	ZrO_2_, Na_2_B_4_O_7_, Mg	Molten-salt	1200	180	Ar	
[39]	ZrB_2_	KBF_4_, K_2_ZrF6, Al	Molten-salt	800	120	Ar	Plate-like
[38]	ZrB_2_	ZrOCl_2_·8H_2_O, Na_2_B_4_O_7_·10H_2_O, C_12_H_22_O_11_	Molten-salt	1400	240	Ar	Rod-like
[16]	ZrB_2_	ZrOCl_2_-8H_2_O, H_3_BO_3_, Chitosan Glutaraldehyde, raw ZrB_2_	Microwave	1320	60	Ar	Columnar
[5]	ZrB_2_-SiC	ZrOCl_2_·8H_2_O, H_3_BO_3_, C_6_H_12_O_6_·H_2_O, C_6_H_8_O_7_·H_2_O, C_2_H_6_O_2_	Microwave	1300	180	Ar	
[15]	ZrB_2_-SiC	ZrSiO_4_, B_2_O_3_, C	Microwave	1300	180	Ar	
This work	ZrB_2_-SiC	ZrO_2_, B_4_C, C	Microwave and Molten-salt	1200	20	Ar	Rod-like
